# Radial Pressure Pulse and Heart Rate Variability in Heat- and Cold-Stressed Humans

**DOI:** 10.1155/2011/751317

**Published:** 2010-11-14

**Authors:** Chin-Ming Huang, Hsien-Cheh Chang, Shung-Te Kao, Tsai-Chung Li, Ching-Chuan Wei, Chiachung Chen, Yin-Tzu Liao, Fun-Jou Chen

**Affiliations:** ^1^School of Chinese Medicine, China Medical University, Taichung City 40402, Taiwan; ^2^Graduate Institute of Biostatistics, China Medical University, Taichung City 40402, Taiwan; ^3^Department of Information and Communication Engineering, Chaoyang University of Technology, Taichung County 412, Taiwan; ^4^Department of Bioindustrial Mechatronics Engineering, National ChungHsing University, Taichung 402, Taiwan; ^5^Institute of Integrated Medicine, China Medical University, Taichung City 40402, Taiwan

## Abstract

This study aims to explore the effects of heat and cold stress on the radial pressure pulse (RPP) and heart rate variability (HRV). The subjects immersed their left hand into 45°C and 7°C water for 2 minutes. Sixty healthy subjects (age 25 ± 4 yr; 29 men and 31 women) were enrolled in this study. All subjects underwent the supine temperature measurements of the bilateral forearms, brachial arterial blood pressure, HRV and RPP with a pulse analyzer in normothermic conditions, and thermal stresses. The power spectral low-frequency (LF) and high-frequency (HF) components of HRV decreased in the heat test and increased in the cold test. The heat stress significantly reduced radial augmentation index (AIr) (*P* < .05), but the cold stress significantly increased AIr (*P* < .01). The spectral energy of RPP did not show any statistical difference in 0 ~ 10 Hz region under both conditions, but in the region of 10 ~ 50 Hz, there was a significant increase (*P* < .01) in the heat test and a significant decrease in the cold test (*P* < .01). The changes in AIr induced by heat and cold stress were significantly negatively correlated with the spectral energy in the region of 10 ~ 50 Hz (SE_10−50 Hz_) but not in the region of 0 ~ 10 Hz (SE_0−10 Hz_). The results demonstrated that the SE_10−50 Hz_, which only possessed a small percentage in total pulse energy, presented more physiological characteristics than the SE_0−10 Hz_ under the thermal stresses.

## 1. Introduction

The radial pressure pulse (RPP), which is derived from the heart's pumping action and transmitted from the heart to the end of the radial artery, is regulated by sympathetic nerve activity (SNA). The RPP conveys much information about the physiology of the cardiovascular system. For example, it carries the effects of meditation on the cardiovascular system [1]. Dating back two thousand years ago, the Chinese doctors placed their fingertips on patients' wrists to palpate the pulse property for diagnosis. Nowadays, a study has confirmed the quantitative characteristics of the pulse signals from RPP [2]. By RPP, we can evaluate the augmentation index, elasticity of the artery, and pulse wave velocity among others. Studies have indicated that the radial augmentation index (AIr) and radial diastolic augmentation (DAI) can reflect the left ventricular load [3]. Peripheral blood pressure pulse transmits along the arterial tree and is reflected to become a retrograde wave when the pulse encounters the resistance of the arterial wall. As a result, increasing the peripheral resistance may induce the higher degree of reflected waves that then results in higher amplitude of AIr [4]. The peripheral vascular vasomotion is generated and modulated by the SNA [5]. Studies have demonstrated that lowering the temperature induces peripheral vasoconstriction and decreases vasomotion; these responses are reversed if the temperature is raised [6].

The cold pressor test was to immerse the subject's hand into 1 ~ 7°C water for 1 ~ 6 minutes, which resulted in high-stressed responses including a rise in blood pressure and heart rate [7, 8]. Furthermore, the studies have found that either environmental stress or cold stress can affect immune function [9, 10]. The spectral analysis of HRV is a quantitative tool for evaluating the autonomic nervous system of the heart [11–13]. The high-frequency component (HF, 0.15 ~ 0.4 Hz) of HRV reflects cardiac vagal nervous activity, and the low-frequency component (LF, 0.04 ~ 0.15 Hz) is mediated by both cardiac vagal and SNA; therefore, the ratio of low- to high-frequency component (LF/HF) is an index of cardiac sympathovagal balance. The HF power (HF%), normalized to the total spectral power, is regarded as an indicator of parasympathetic activity [14]. The spectral analysis of HRV is often used to assess the effects of heat and cold tests [15]. Besides the evaluating AIr in the time domain, the spectra transformed into frequency domain are mostly used for the periodic waves to represent the dynamics carried in the waves by obtaining the sums of the spectral energy (SE). In normal individuals, the spectral energy within 0 ~ 10 Hz (SE_0−10Hz_) took more than 99.1% of the total energy of the radial pressure waveforms, with less than 0.9% of energy within 10 ~ 50 Hz (SE_10−50Hz_), and it had large variations above 10 Hz for patients who had acute illness or under metabolic stress [16, 17]. The spectral harmonic energy ratio has been studied to reveal the state of blood circulation [18]. Therefore, RPP always exhibits some specific characteristics in the spectral domain. However, the spectral characteristics of RPP for humans under heat and cold stress have not been well studied. This study aims to explore the spectral and time-domain effects on the RPP and HRV in heat and cold stress.

## 2. Methods

### 2.1. Subjects

Sixty healthy subjects (29 men and 31 women) participated in the study. The mean age in men was 25.1 ± 4.0 yr, and that in women was 24 .7 ± 4.3 yr. The protocol and informed consent were approved by the IRB in China Medical University Hospital (DMR97-IRB-191). To exclude participants with acute illness, all subjects underwent a detailed medical examination including a medical history survey and a physical examination. A written informed consent was obtained from each participant before the experiment. Consumptions of caffeinated and alcoholic beverages as well as smoking were forbidden for a period of 24 hours before the test.

### 2.2. Testing Protocol

**Figure 1:**
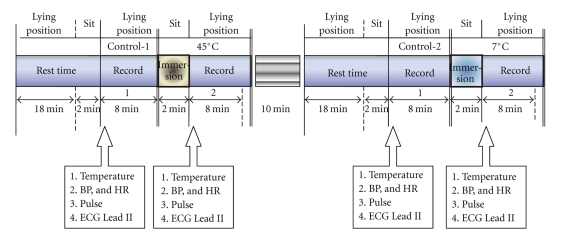
The experimental procedure is as shown. Initially, the subject rested in the supine position for 18 minutes and then sat up for 2 minutes. Subsequently, the subject lay down again for the measurement. Afterward, the subject sat up and immersed his/her left hand into 45°C water for 2 minutes, and the same variables were measured again. After the heat test, the subject rested another 10 minutes, and the prior procedure was repeated for the immersion of 7°C water.

All subjects participated in the two tests at normothermic conditions by immersing their left hands into 45°C water and 7°C water. The experimental procedures are shown in Figure 1. During the experiment, the room temperature was maintained at about 26 ~ 27°C. The subjects were instructed to lie down in the supine position and relax for 20 minutes. During the last 2 minutes, the subjects sat up and subsequently lay down again to take the measurements including the temperatures on their both forearms, blood pressure (BP), heart rate (HR), 5-minute ECG, and radial pulse at the right wrist. These data were used as the variables for control-1. Afterward, the subjects immersed their left hands into 45°C water for a period of 2 minutes. After removing hands out from the water, the subjects were gone through the same measurement procedures. When the heat test was finished, the subjects rested for 10 minutes and then repeated the prior procedures with the water temperature being changed from 45°C to 7°C. The measurements before the 7°C water immersing were recorded as control-2 variables.

### 2.3. Measurements

#### 2.3.1. Skin Temperature

An infrared thermometer (Omni-Nure TE-HBI, Constant Healthcare Technology Co., Ltd., Taiwan) was used to record the skin temperature on the inner sides of both forearms 3 cm below the elbow joint by slightly touching the skin.

#### 2.3.2. Hemodynamics

BP (systolic and diastolic blood pressure) and heart rate were measured by the Panasonic Diagnostic Upper Arm Blood Pressure Monitor (Matsushita Electric Works, Ltd., Osaka, Japan) through a cuff wrapped around the upper left arm.

#### 2.3.3. Pulse and Analysis

**Figure 2:**
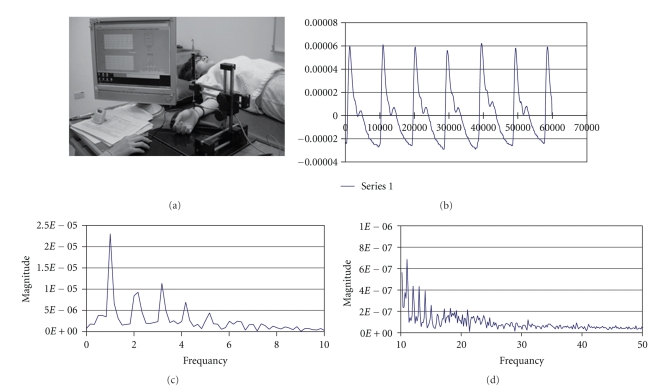
Pulse measurement and analysis. The pulse was measured (a) on the right radial artery with the subject in the supine position to obtain, (b) a typical radial pressure pulse waveform, and the electrical pulse signal was digitized and analyzed to acquire the corresponding spectrogram, (c) the 0 ~ 10 Hz band, and (d) the 10 ~ 50 Hz band.

**Figure 3:**
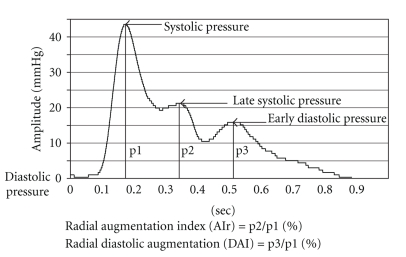
A typical radial pressure pulse waveform in this experiment. Measures of morphology include AIr, calculated as (late systolic pressure (p2)-diastolic pressure)/(systolic pressure (p1)-diastolic pressure) × 100%, and DAI was calculated as (early diastolic pressure (p3)-diastolicpressure)/(systolic pressure (p1)-diastolic pressure) × 100%. Diastolic pressure was zero in radial pressure pulse waveform.

The right RPP was recorded by the Pulse analyzer (designed by China Medical University, Taiwan) consisting of a high-fidelity pressure sensor and a stable X-Y-Z axial moveable framework. The examinations were carried out with each subject lying in the supine position, as shown in Figure 2(a). When the sphygmogram showed the greatest amplitude, as in Figure 2(b), the RPP was considered suitable to be recorded. The electrical pulse signal from the sensor was digitized and fed into a computer for processing through fast Fourier transformation to obtain the sums of the SE_0−10Hz_ and SE_10−50Hz_. The corresponding spectrogram of 0 ~ 10 Hz and 10 ~ 50 Hz bands are shown in Figures 2(c) and 2(d). In Figure 3, AIr was calculated as (late systolic pressure – diastolic pressure)/(systolic pressure – diastolic pressure) × 100%, and DAI was calculated as (early diastolic pressure – diastolic pressure)/(systolic pressure – diastolic pressure) × 100% [3].

#### 2.3.4. HRV and Analysis

The HRV analyzer (designed by China Medical University, Taiwan) with one-channel electrocardiograph (Lead II) was used to record the surface ECG from the subjects in the lying position for five minutes. The analogue ECG signals were immediately converted into digital signals. R-R intervals were measured after the R waves were detected. The ECG signals contained abnormal complexes or artifacts were discarded. Parameters of the HRV spectral analysis were computed from the sequence of normal R-R intervals by means of the fast Fourier transformation. LF (0.04 ~ 0.15 Hz) and HF (0.15 ~ 0.5 Hz) were determined by integrating the power spectrum density in the respective frequency range. Then, the normalized unit, HF% = 100 × HF/(LF + HF), and LF/HF were calculated.

### 2.4. Statistical Methods

In addition to the calculation of mean ± SD, the paired *t*-test was used to compare the temperature difference between two forearms in the baseline condition, and the variance changed from normothermic condition to the thermal stress condition. Pearson's correlation analysis was used to study the variables between the spectral energy of RPP and AIr induced by heat and cold. All tests were two sided; *P* < .05 was taken as significant. Statistical analysis was performed by the Statistical software SPSS 15.0 for Windows (SPSS Inc.).

## 3. Results

All subjects completed the experimental trials and measurements. The responses of physiological variables, HRV and RPP, are presented in Table 1. There was significant difference in the baseline temperatures of the left and right forearms (*P* = .000043).

### 3.1. Physiological Variables

**Figure 4:**
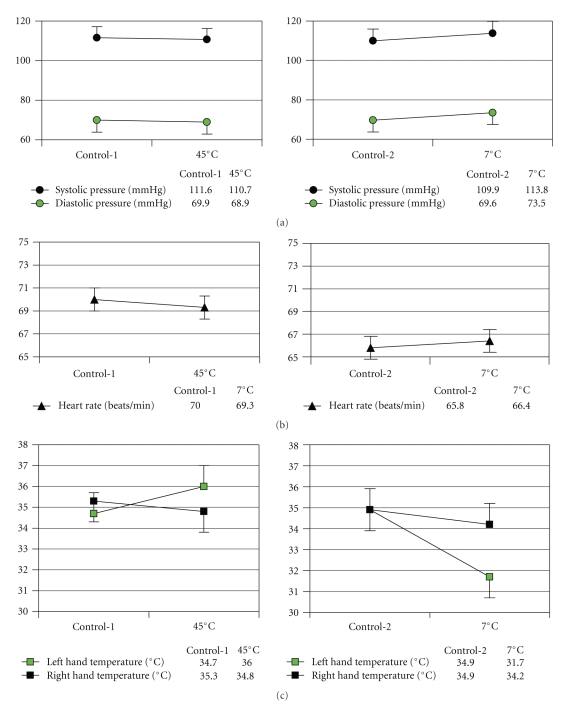
Effects of thermal stresses on the physiological functions. (a) systolic and diastolic pressure decreased after heat stress and increased after cold stress, (b) heart rate decrease after heat stress and increase after cold stress, and (c) left hand temperature increased and right hand temperature decreased after heat stress, and temperatures of both hands decreased after cold stress. ‡: left-hand temperature versus right-hand temperature in baseline control and *P* < .01; **P < .01 compared with the control.

Changes in physiological variables are shown in Figure 4. In the heat test, the mean heart beat slowed down, and the systolic and diastolic pressure decreased; however, these responses were not significantly different with that in control-1. The temperature in the left hand significantly increased than that in the normothermic control-1 (*P* < .01), and it significantly decreased in the right hand (*P* < .01). In the cold test, systolic and diastolic pressure significantly increased (*P* < .01), and temperatures in both hands significantly decreased (*P* < .01).

### 3.2. Heart Rate Variability (HRV)

**Figure 5:**
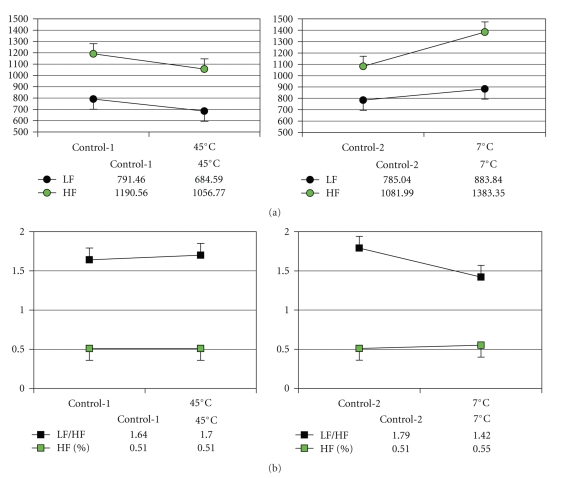
Effects of thermal stresses on the HRV. (a) The integrations of HF and LF decreased after heat stress and increased after cold stress, and (b) LF/HF ratio increased after heat stress and decreased after cold stress, and there were no changes in HF% after heat stress and increases after cold stress, *P < .05 compared with the control.

Changes in the spectral components of HRV are shown in Figure 5. The power spectrum of HRV revealed a decreased power for the LF and HF components after heat stress and an increased power after cold stress. These changes were not significantly different in this experiment. There was no significant difference in LF/HF either, but the HF% significantly increased by comparing the 7°C water immersion with normothermic control-2 (*P* < .05).

### 3.3. Radial Pressure Pulse

**Figure 6:**
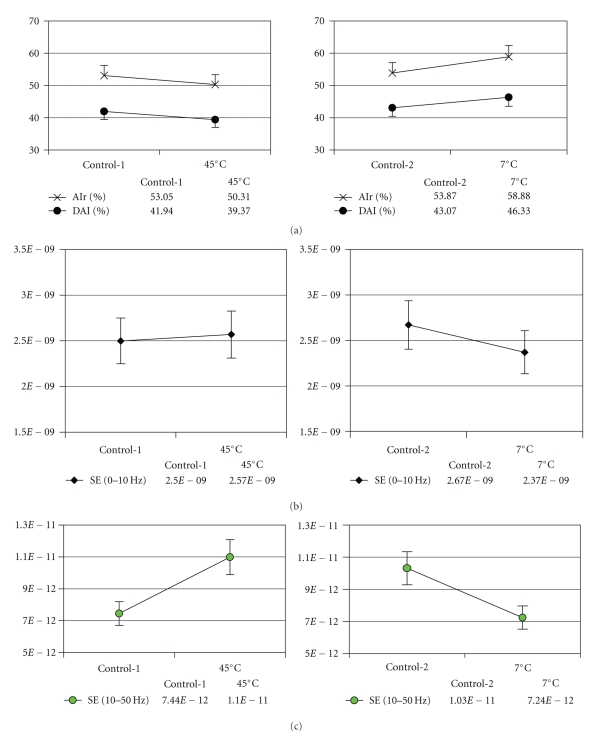
Effects of thermal stresses on the radial pressure pulse. (a) AIr and DAI decreased after heat stress and increased after cold stress, (b) Spectral energy (SE) of 0 ~ 10 Hz and 10 ~ 50 Hz increased after heat stress and decreased after cold stress. (c) *P < .05 compared with the control; **P < .01 compared with the control.

Changes in the parameters of RPP are shown in Figure 6. There were no statistical differences in the amplitudes of p1, p2, or p3 in the heat test nor in those of p2 or p3 in the cold test, but there was a statistical decrease in the p1 in the cold test (*P* < .05). The heat test significantly reduced the AIr and DAI (*P* < .05), but the cold test significantly increased the AIr (*P* < .01) and DAI (*P* < .05). In SE_0−10Hz_, there was no statistical difference in the heat or cold test. However, SE_10−50Hz_ significantly increased (*P* < .01) in the heat test and significantly reversed in the cold test (*P* < .01). Pearson's correlation analysis demonstrated that the changes in AIr induced by the heat and cold stress were significantly negatively correlated with the induced changes in SE_10−50Hz_ (Pearson's correlation coefficients: *r* = −0.289, *P* = .02519; *r* = −0.315, *P* = .01424, resp.), but the changes in AIr were not significantly correlated with that in SE_0−10Hz_ (Pearson's correlation coefficients: *r* = −0.112, *P* = .39482; *r* = −0.139, *P* = .2884, resp.).

## 4. Discussion

The significant findings of this study are that the spectral energy of radial artery can be altered by heat and cold stress from the skin surface, and these changes did not converge in lower frequency bands, 0 ~ 10 Hz, but in higher frequency bands, 10 ~ 50 Hz, and the variables induced by heat and cold in AIr and SE_10−50Hz_ had significantly negative correlation. The results demonstrate that the SE_10−50Hz_, which only possesses a small percentage in total pulse energy, presents more physiological characteristics than the SE_0−10Hz _in the thermal stresses.

In baseline condition, the mean skin temperature (*T*_sk_) of the right hand was significantly higher than that of left hand. The result is consistent with the study by Luis Rodrigues et al. (1998), which revealed that the right forearm was more motoric than the left one [19]. It increased during the heat stress while decreased under cold stress (Figure 4). Although the heat and cold stimulation were carried out only on the left hand, the *T*_sk_ changes of both hands showed significant difference. The heat stimulation in the left hand induced the *T*_sk_ to be increased significantly in the hand itself whereas the *T*_sk_ in the right hand significantly decreased. The physiological study has shown that the microcirculation is a functional system by the reflex of the automatic feedback system. For example, the skin blood flow is reflectively modulated by the vasodilator and vasoconstrictor in response to the change in skin temperature, and the subcutaneous vasomotor activity is synchronous on bilateral limbs [20, 21]. Therefore, the heat stress induced the vasodilatation of the left hand by directly contacting with hot water, and at the same time, it resulted in the loss of dermal heat in the right hand synchronously. The result reveals that the subcutaneous thermoregulation is systemic after undergoing heat and cold stress. This result is similar to the study by Bozdemir et al. (1998) that cold stress causes systemic vasomotor changes [22]. Figure 5 presents that HF% is a significant increase after 7°C immersion, and both LF and HF increase in the cold test but decrease in the heat test, which reveal that skin surface cooling can augment the cardiac sympathetic and vagal nervous activity, but the skin surface heating is reversed. The responses in this experiment are consistent with prior study by Kinugasa and Hirayanagi (1999) [23].

Skin surface cooling induces peripheral vasoconstriction and decreases vasomotion. According to the Poiseuille's law, the small decrease in the lumen of small arteries and arterioles may significantly increase blood pressure and peripheral vascular resistance, which results in an increase of reflection wave. In contrast, heat induces peripheral vasodilatation and increases vasomotion to decrease blood pressure and reflection wave [6, 24, 25]. In Figures 4 and 6, the heat stress decreases systolic and diastolic pressure and significantly reduces the AIr and the DAI, which are associated with peripheral vasodilatation [3]. The cold stress significantly increases the systolic and the diastolic pressure and significantly increases the AIr and the DAI, which are associated with peripheral vasoconstriction [26]. Through these results, it is comprehended that the heat decreases the left ventricular load whereas the cold increases the left ventricular load [3]. Figure 6 presents that the SE_0−10Hz_ showed no statistical change in both experiments. In the heat test, the SE_10−50Hz_ significantly increased, but the response was significantly reversed in the cold test. In cardiovascular physiology, the vascular smooth muscles are innervated by nerve fibers and exposed to recurrent oscillated stimuli; thus, the motion of vessels is influenced by nerve activity. Both heat and cold stresses can significantly increase peripheral SNA [7, 27]. Most of the sympathetic nerve discharge is between 2 and 6 Hz, and it would be increased to 50 Hz during acute stress, which maximizes the arterial and arteriolar tension [28]. The increased impedance of vessels is proportional to the frequency. Such a variation in the tension of vascular wall will lead to the fluctuation of blood pressure pulse with the higher natural frequency response [29]. Hence, it is inferred that the tension fluctuation of the time-domain pressure pulse may correspond to the fluctuation of a relatively higher frequency band (10 ~ 50 Hz). The result that SE_10−50Hz_ is negatively correlated to AIr is consistent with the theory.

The RPP was measured at the end of the radial artery, which is adjacent to the small arteries and arterioles. Hence, the RPP has its own blood pressure and receives the higher fluctuation frequency of blood flow pressure from the retrograde waves of the small arteries and arterioles. Energy dissipation of blood flow is necessary to overcome peripheral vascular resistance [30]. The reductions of peripheral vascular lumen and vasomotion under cold stress induce energy dissipation and increase the amplitude of reflection waves; subsequently, higher frequency energy, SE_10−50Hz_, is decreased. In contrast, the increases of peripheral vascular dilatation and vasomotion in heat stress decrease energy dissipation and reduce the amplitude of reflection waves; higher frequency energy, SE_10−50Hz_, is increased subsequently.

In conclusion, our study demonstrates the following results. (1) The skin temperature of the right hand is significantly higher than that of the left hand. The change of *T*_sk_ induced by local skin heat and cold on the left hand is systemic. (2) The heat reduces AIr and DAI, which indicates a decrease of the left ventricular load; the cold increases AIr and DAI, which indicates an increase of the left ventricular load. (3) The SE_10−50Hz_, which only possesses a small percentage in total pulse energy, presents more physiological characteristics than the SE_0−10Hz _under thermal stresses.

## Figures and Tables

**Table 1:** The experimental variables after 45°C and 7°C water immersion.

	Heating experiment			Cooling experiment		
	Control-1	45°C	*P*-value	Control-2	7°C	*P*-value
HR, beats/min	70.3 ± 10.5	69.5 ± 9.8	.157	65.9 ± 9.8	66.6 ± 10.2	.343
S_P, mmHg	111.6 ± 6.9	110.7 ± 7.7	.263	110.0 ± 9.2	113.8 ± 9.4	2.21*E*-5**
D_P, mmHg	69.8 ± 6.7	68.8 ± 7.0	.132	69.8 ± 6.5	73.6 ± 5.9	9.93*E*-7**
L_H temp, °C	34.8 ± 1.8^‡^	36.0 ± 1.4	1.09*E*-9**	34.9 ± 1.7	31.6 ± 1.3	1.84*E*-24**
R_H temp, °C	35.3 ± 1.6^‡^	34.8 ± 1.7	1.82*E*-4**	34.9 ± 1.8	34.2 ± 2.0	1.77*E*-5**
HRV, LF	791.46 ± 1117.96	684.59 ± 599.50	.4314	785.04 ± 793.48	883.84 ± 1003.82	.334
HRV, HF	1190.56 ± 2490.21	1056.77 ± 1519.63	.443	1081.99 ± 1394.05	1383.35 ± 2329.98	.066
HRV, LF/HF	1.64 ± 2.16	1.70 ± 2.02	.793	1.79 ± 2.27	1.42 ± 1.94	.076
HRV, HF%	0.51 ± 0.20	0.51 ± 0.23	.763	0.51 ± 0.23	0.55 ± 0.21	.033*
SE, 0–10 Hz	2.50*E*-09 ± 1.07	2.56*E*-09 ± 1.19	.669	2.69*E*-09 ± 1.22	2.36*E*-09 ± 1.61	.054
SE, 10–50 Hz	7.51*E*-12 ± 5.91	11.01*E*-12 ± 7.58	4.05*E*-5**	10.40*E*-12 ± 8.10	7.28*E*-12 ± 7.16	.005**
p1, mmHg	35.98 ± 7.67	36.92 ± 8.75	.384	37.63 ± 9.51	34.43 ± 11.55	.012*
p2, mmHg	18.9 ± 5.82	18.37 ± 6.33	.483	19.82 ± 5.66	19.72 ± 7.58	.904
p3, mmHg	14.98 ± 4.74	14.61 ± 5.2	.534	16.11 ± 4.92	15.81 ± 6.81	.670
AIr, %	53.05 ± 14.08	50.31 ± 14.4	.046*	53.87 ± 14.27	58.88 ± 15.77	3.74*E*-4**
DAI, %	41.94 ± 10.65	39.37 ± 8.85	.028*	43.07 ± 8.92	46.33 ± 12.52	.011*

Values are means ± SD (*n* = 60). HR: heart rate; S_P: systolic pressure; D_P: diastolic pressure; L_H temp: left-hand temperature; R_H temp: right-hand temperature; SE: Spectral energy. ‡: left-hand temperature versus right-hand temperature in baseline control and *P* < .01; *P < .05 compared with the control for each experiment; **P < .01 compared with the control for each experiment. p1: systolic pressure, p2: late systolic pressure, p3: early diastolic pressure.

## References

[1] Liu C-Y, Wei C-C, Lo P-C (2009). Variation analysis of sphygmogram to assess cardiovascular system under meditation. *Evidence-Based Complementary and Alternative Medicine*.

[2] Jeon YJ, Kim JU, Lee HJ A clinical study of the pulse wave characteristics at the three pulse diagnosis positions of Chon, Gwan and Cheok.

[3] Munir S, Jiang B, Guilcher A (2008). Exercise reduces arterial pressure augmentation through vasodilation of muscular arteries in humans. *American Journal of Physiology*.

[4] Safar ME, Lacolley P (2007). Disturbance of macro- and microcirculation: relations with pulse pressure and cardiac organ damage. *American Journal of Physiology*.

[5] Segal SS (2005). Regulation of blood flow in the microcirculation. *Microcirculation*.

[6] Kellogg DL (2006). In vivo mechanisms of cutaneous vasodilation and vasoconstriction in humans during thermoregulatory challenges. *Journal of Applied Physiology*.

[7] Victor RG, Leimbach WN, Seals DR, Wallin BG, Mark AL (1987). Effects of the cold pressor test on muscle sympathetic nerve activity in humans. *Hypertension*.

[8] Mitchell LA, MacDonald RAR, Brodie EE (2004). Temperature and the cold pressor test. *Journal of Pain*.

[9] Vojdani A, Lambert J The role of Th17 in neuroimmune disorders: target for CAM therapy. Part II.

[10] Jiang X-H, Guo S-Y, Xu S (2004). Sympathetic nervous system mediates cold stress-induced suppression of natural killer cytotoxicity in rats. *Neuroscience Letters*.

[11] Arai Y-CP, Ushida T, Matsubara T The influence of acupressure at extra 1 acupuncture point on the spectral entropy of the EEG and the LF/HF ratio of heart rate variability.

[12] Chien L-W, Lin M-H, Chung H-Y, Liu C-F Transcutaneous electrical stimulation of acupoints changes body composition and heart rate variability in postmenopausal women with obesity.

[13] Lee H-J, Chae Y, Park H-J, Hahm D-H, An K, Lee H (2009). Turo (Qi dance) training attenuates psychological symptoms and sympathetic activation induced by mental stress in healthy women. *Evidence-Based Complementary and Alternative Medicine*.

[14] Task Force of the European Society of Cardiology and the North American Society of Pacing and Electrophysiology (1996). Heart rate variability: standards of measurement, physiological interpretation and clinical use. *Circulation*.

[15] Yamazaki F, Sone R (2000). Modulation of arterial baroreflex control of heart rate by skin cooling and heating in humans. *Journal of Applied Physiology*.

[16] Wei LY, Lee CT, Chow P (1984). A new scientific method of pulse diagnosis. *American Journal of Acupuncture*.

[17] Imperial-Perez F, McRae M (2002). Protocols for practice: applying research at the bedside. *Critical Care Nurse*.

[18] Huang C-M, Wei C-C, Liao Y-T, Chang H-C, Kao S-T, Li T-C Developing the effective method of spectral harmonic energy ratio to analyze the arterial pulse spectrum.

[19] Rodrigues L, Pereira LM (1998). Basal transepidermal water loss: right/left forearm difference and motoric dominance. *Skin Research and Technology*.

[20] Braverman IM (2000). The cutaneous microcirculation. *Journal of Investigative Dermatology Symposium Proceedings*.

[21] Kamijo Y-I, Lee K, Mack GW (2005). Active cutaneous vasodilation in resting humans during mild heat stress. *Journal of Applied Physiology*.

[22] Bozdemir H, Sarica Y, Demirkiran M (2002). The effects of cold stress test on vasomotor tonus in normal controls. *Neurology India*.

[23] Kinugasa H, Hirayanagi K (1999). Effects of skin surface cooling and heating on autonomic nervous activity and baroreflex sensitivity in humans. *Experimental Physiology*.

[24] Schiffrin EL (2004). Remodeling of resistance arteries in essential hypertension and effects of antihypertensive treatment. *American Journal of Hypertension*.

[25] Rowell LB (1977). Reflex control of the cutaneous vasculature. *Journal of Investigative Dermatology*.

[26] Edwards DG, Roy MS, Prasad RY (2008). Wave reflection augments central systolic and pulse pressures during facial cooling. *American Journal of Physiology*.

[27] Cui J, Sathishkumar M, Wilson TE, Shibasaki M, Davis SL, Crandall CG (2006). Spectral characteristics of skin sympathetic nerve activity in heat-stressed humans. *American Journal of Physiology*.

[28] Kenney MJ (1994). Frequency characteristics of sympathetic nerve discharge in anesthetized rats. *American Journal of Physiology*.

[29] Kinefuchi Y, Fukuyama H, Suzuki T, Kanazawa M, Takiguchi M (1999). Development of a new catheter-tip pressure transducer. *Tokai Journal of Experimental and Clinical Medicine*.

[30] Agabiti Rosei E, Rizzoni D (2005). Pathophysiology and clinical meaning of small resistance artery remodeling. *Current Hypertension Reports*.

